# Systems of care for ambulatory management of decompensated heart failure

**DOI:** 10.3389/fcvm.2024.1350846

**Published:** 2024-02-21

**Authors:** Narotham Badrish, Stuart Sheifer, Carolyn M. Rosner

**Affiliations:** ^1^ Department of Cardiology, Inova Schar Heart and Vascular, Falls Church, VA, United States; ^2^ Department of Cardiology, Virginia Heart, Falls Church, VA, United States

**Keywords:** heart failure, decompensated heart failure, ambulatory, systems of care, remote monitoring

## Abstract

Heart failure (HF) represents a worldwide health burden and the annual per patient cost to treat HF in the US is estimated at $24,383, with most of this expense driven by HF related hospitalizations. Decompensated HF is a leading cause for hospital admissions and is associated with an increased risk of subsequent morbidity and mortality. Many hospital admissions for decompensated HF are considered preventable with timely recognition and effective intervention.Systems of care that include interventions to facilitate early recognition, timely and appropriate intervention, intensification of care, and optimization to prevent recurrence can help successfully manage decompensated HF in the ambulatory setting and avoid hospitalization.

## Introduction

Heart failure (HF) represents a massive health burden worldwide, with approximately 6 million adults in the United States carrying the diagnosis (1). As the annual per patient cost to treat HF is estimated at $24,383, the financial impact is significant, and most of this expense is due to HF related hospitalizations (2). Decompensated HF is a leading cause for hospital admissions and carries negative prognostic implications, as hospitalization is associated with an increased risk of subsequent morbidity and mortality (3–5).

**Figure 1:**
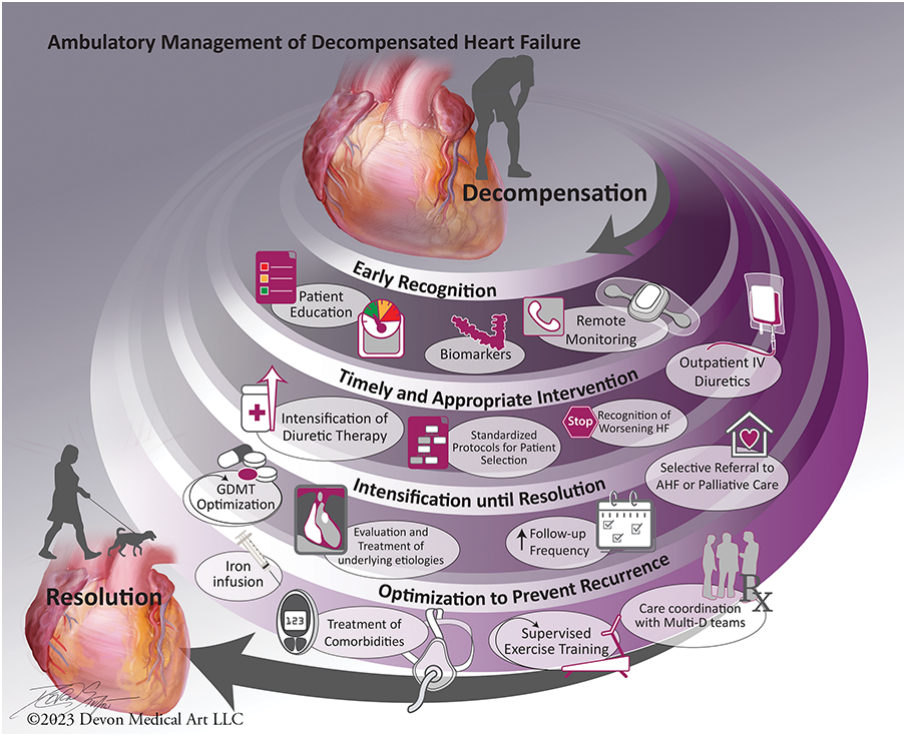
Main concepts in systems of care for ambulatory management of acute decompensated heart failure.

Many hospital admissions for decompensated HF are considered preventable with timely recognition and effective intervention (6). Interventions targeting preventing hospitalization for decompensated HF are increasingly patient-centered, with both an episodic focus and longitudinal perspective (7–9). Four key concepts are consistently associated with successful systems of care for ambulatory management of decompensated HF: (1) Early recognition of decompensation; (2) Timely and appropriate intervention to address decompensation; (3) Intensification of care until stabilization; and (4) Optimization to prevent recurrence (Figure 1).

We will present current evidence for interventions corresponding with each of these elements, the residual clinical challenges, and future directions for care improvement (Table 1).

**Table 1:** Concepts in systems of care for ambulatory management of decompensated heart failure.

Component of care	Associated interventions	Future directions
Early recognition	• Patient education and self-monitoring (18, 19) • Remote monitoring ○ Invasive (20–24) ○ Non-invasive (17, 19) • Biomarkers (17, 19, 30, 32)	• Improved identification of impending decompensation via multiparameter models • Increased EMR integration of data from wearable and remote monitoring devices • Integration of artificial intelligence • Equitable access to remote and wearable devices • Increased utilization of point of care testing
Timely and appropriate intervention	• Assessment and treatment as soon as decompensation is recognized (38) • Recognition of worsening HF (10) • Standardized protocols to identify risk and guide appropriate patient selection for OP management, particularly discharge from emergency department (35) • Intensification of diuretic regimens (34, 37) • Outpatient IV diuretics (38, 40–44)	• Home treatment: ○ In home via provider ○ SQ diuretics (self-administered) • Outpatient IV diuretics widely available • Widespread use of algorithms to identify low risk patients with HF who can be managed safely in the ambulatory setting
Intensification of care until stabilization	• Increased follow up frequency (35, 44) • Assessment and treatment of underlying etiologies and triggers (arrhythmias, ischemia) (45–50) • Selective referral to AHF and palliative care (51) • GDMT optimization (32, 52–55)	• Timing and patient selection for the ideal use of coronary revascularization • Timing and patient selection for the ideal use of atrial and ventricular ablation • Continued work on strategies to increase GDMT utilization
Optimization to prevent recurrence	• Utilization of multi-D teams with pharmacy, nursing, and social work (63, 64) • Iron infusion (57–59, 65, 66) • Supervised exercise training (60–62, 67) • Optimization of comorbidities (sleep apnea, HTN, DM) (17) • Treatment of depression (71, 72)	• Addressing barriers to care and social determinants of health in HF care • Integration of care teams • Holistic approach to HF care • Focus on value based HF care

### Early recognition of decompensation

The first key element of a successful system of care for ambulatory management of decompensated HF is early recognition of an episode of active or impending decompensation. The concept of “decompensated” HF describes an exacerbation or abrupt worsening of symptoms in individuals with pre-existing HF (10). While typically considered a rapid onset of severe symptoms requiring immediate attention and frequently resulting in hospitalization, decompensation can also occur gradually (10). Symptoms of decompensation are often noted after a patient has been in the state for several days and prompt recognition is critical to facilitate appropriate treatment (11). Interventions shown to facilitate early recognition of decompensated HF include patient education, clinician driven remote patient management, remote monitoring, and biomarkers.

Patients themselves play a critical role in recognizing and responding to indicators of decompensation, and they should be routinely taught to check weight, heart rate (HR) and blood pressure (BP) regularly and report significant alterations to their healthcare provider (12). Several studies of this approach produced variable results in improvements in clinical outcomes and quality of life (QOL), but may have been confounded by poor adherence and lack of clear comprehensive management algorithm (12–15). In contrast, the TIM-HF2 trial showed reduction in the number of days hospitalized and all-cause mortality in patients with HF with EF <45%, using a structured approach to remote patient management, and self-care and monitoring that are widely used and incorporated in HF management guidelines (16–19).

Advances in technology have led to increased utilization of medical devices to improve early detection of alterations in health (11, 18). The CardioMEMS™ device is approved by the US Food and Drug Administration to wirelessly monitor pulmonary artery (PA) pressure and HR in patients with HF who were recently hospitalized or have elevated naturetic peptides (NP) with NYHA class II or III symptoms and is an important tool for early detection of decompensation (20). The readings from CardioMEMS™ devices are transmitted to patients' clinicians who can adjust HF therapy, specifically intensify diuretic regimens, as needed and often before symptoms of decompensation are present. In the CHAMPION trial, remote monitoring via PA pressure monitoring was associated with a 37% reduction in HF hospitalizations over 15 months for patients with symptomatic HF with preserved ejection fraction (HFpEF) and HF with reduced ejection fraction (HFrEF), as compared to usual care (21, 22). Some of the benefits of remote PA pressure monitoring are that it requires minimal effort from patients, yielding a high rate of adherence with daily pressure transmissions in trials, and facilitates appropriate and safe adjustment of medical therapy and guideline directed medical therapy (GDMT) optimization (21–23). In addition to the clinical benefits, the GUIDE-HF trial showed that the CardioMEMS device was associated with improved QOL (24). Some potential challenges in implementation include the need for adequate internet for data transmission, clinic infrastructure required to manage often large volumes of readings from patients in a timely and effective way, and occasional need to repeat right heart catheterization and recalibration to ensure accurate readings (25, 26).

Most contemporary implantable defibrillators and pacemakers include technology that evaluate physiologic variables and can provide diagnostic information to aid in management of patients with HF (20). Data provided from these devices includes heart rate variability, activity, thoracic impedance, as well as atrial and ventricular arrhythmias (20). The use of multiple parameters from these devices has great potential for clinical utility though has had limited evidence supporting improved HF outcomes, likely related to lack of standardization in the response to the data (27–29).

Lastly, biomarkers are another tool for early detection of decompensation with established utility in diagnosing HF and assessing prognosis (17, 30). The most frequently utilized biomarkers in HF care are NP, which have excellent specificity for diagnosing HF (30). Beyond their role in establishing diagnosis, the Val-HeFT study demonstrated that changes in NT-proBNP values over time were closely associated with changes in prognosis, including prediction of mortality (31). While GUIDE-IT did not demonstrate benefit in utilizing NP to guide HF therapies, more recently, STRONG-HF demonstrated that a strategy of rapid up titration of GDMT, as compared to those in the usual care, resulted in more patients with HF reaching target doses of these therapies and also lower NT-proBNP levels, and also incorporated modifications to GDMT titration schedule based on elevated NT-proBNP levels (32, 33).

### Timely and appropriate intervention to address decompensation

Once a patient with HF shows signs and symptoms of decompensation, it is imperative to act quickly to implement the appropriate treatment (34). A key factor in determining if outpatient management is safe and likely to be effective involves determining if the episode is consistent with “decompensated HF” or “worsening HF”. This distinction has been a recent point of focus in care guidance, with decompensating HF connoting the potential for outpatient management, but with worsening HF typically indicating the need for inpatient care (10).

Assessment of end organ function, including renal and hepatic parameters, should be used to augment risk assessment in decompensated HF and to guide management (17). The COACH trial demonstrated that providing decision-making support for clinicians in the emergency department facilitated accurate identification of low risk patients, and that initial treatment there, followed by 30-days of transitional care, yielded a low rate of hospitalization (35). This study contained several key elements to optimize outcomes for patients with acute HF, including a risk-score to guide real-time decision-making, transitional care, and the use of a standardized, dedicated HF clinic (36).

While there are unique differences in the etiologies, treatments, and clinical course of patients with HFrEF as compared to those with HFpEF, both are susceptible to decompensation with volume overload and assessing presence and degree of congestion is an important aspect of determining appropriate interventions (2). Loop diuretics are the mainstay therapy and escalation of oral diuretic regimens should be done as soon as decompensation is identified (37). However, oral diuretics may not be able to achieve adequate diuresis due to interstitial and bowel edema, kidney dysfunction and decreased organ perfusion, and parenteral administration may be needed (38).

Historically, most patients with HF have not had an option for IV diuretics outside of an emergency department or hospital admission (39). However, multiple recent studies have suggested that the use of outpatient IV diuretics is safe and effective in treatment of volume overload and reducing hospitalization (38, 40–44). The main tenets of clinics that offer outpatient IV diuretics includes same or next-day availability, careful patient selection, the use of standardized protocols to guide diuretic dosing, and ability to replace electrolytes and monitor BP to safely achieve large volume diuresis. Other best practices clarified by these studies include close follow up, either with additional visits or return to the primary cardiologist and adjusting outpatient regimens to achieve euvolemia and avoid admission particularly in the next 30 days (38, 40–44).

The use of subcutaneous furosemide for ambulatory management of decompensated HF is a promising recent development, particularly given the challenge of access to outpatient IV diuretics and it overcomes the suboptimal efficacy of oral medications. It has a favorable safety and efficacy profile and represents another outpatient option for the treatment of volume overload (39).

### Intensification of care until stabilization

Intensification of care amidst a HF exacerbation is another important element present in systems of care developed to successfully manage outpatient decompensation. Increasing the frequency of outpatient monitoring, with focus on achieving clinical stabilization and identifying and addressing a reversible or modifiable cause is key in preventing hospitalization and reducing the risk of future exacerbations (35, 38). In addition to ensuring patients with decompensated HF have ongoing appropriate titration of diuretic doses, specific activities in this area include evaluation and treatment of underlying etiologies, selective referral for advanced HF therapies and palliative care consultation and optimization of GDMT.

While an ischemic evaluation is warranted for patients with a new HF diagnosis, the role of coronary revascularization in patients with known HF in a decompensated state is less clear. Some observational studies showed improved outcomes with percutaneous coronary intervention (PCI) in patients with HF and high-grade CAD, however recent data from REVIVED-BCIS2 showed no reduction in all-cause mortality or HF hospitalization for patients with HFrEF related to CAD who underwent PCI, and further investigation is warranted to determine appropriate timing and patient selection (45, 46).

Patients with decompensated HF episode may warrant investigation and treatment of atrial and ventricular arrhythmias, as uncontrolled and persistent arrhythmias can cause HF or trigger decompensation (17, 47). Recent studies have shown that catheter ablation for atrial fibrillation can be superior to medical therapy in select patients with HFrEF in improving survival and reducing HF hospitalizations, and suppression of premature ventricular contractions, either with medication or ablation, way be warranted in the setting of HFrEF (47–50). Additionally, for patients with HF and significant arrhythmias, testing for infiltrative cardiomyopathies should considered (17).

Failure to respond to above mentioned interventions and recurrent HF hospitalizations despite intensifying medical care are important triggers for referral to advanced HF specialists (51). Certain patients may benefit from advanced HF therapies including inotropes, mechanical circulatory support devices, and heart transplantation, and all patients with HF refractory to GDMT merit palliative care consultation to address symptoms and map out goals of care (51).

Lastly and arguably most importantly, intensification of care should focus on prescription and titration of GDMT, which has been repeatedly demonstrated to improve HF symptoms and improve morbidity and mortality (17, 52–55). Despite the weight of evidence, most patients with HFrEF are not currently at optimal doses of all four pillars of GDMT (54). Initiation and rapid up-titration of GDMT has a significant impact on reducing HF hospitalizations, regardless of baseline EF or initial biomarkers, as demonstrated in STRONG-HF (32). This rapid up titration model, wherein most of the GDMT adjustment was delivered in the outpatient setting, has been previously described and encouraged and the positive findings are unsurprising, given the significant and additive benefits of each of the 4-components of GDMT for HFrEF, including reductions in future HF hospitalizations and cardiovascular death (9, 53–56).

### Optimization to prevent recurrence

The final element of systems of care for outpatient management of decompensated HF is a comprehensive focus on optimizing HF care to prevent recurrence. Interventions demonstrated to improve outcomes for patients with HF include iron infusion, supervised exercise training, and treatment of comorbidities (17, 57–62). These activities are often coordinated by multidisciplinary care teams, including social workers and RN navigators, who have been demonstrated to be valuable partners to help address non-clinical barriers to GDMT optimization and facilitate more patients achieving target doses of GDMT (17, 63, 64).

There is a growing interest in the use of IV ferric carboxymaltose (FCM) in the treatment of iron deficiency anemia in patients with HFrEF, with its use associated with improvement in QOL and six-minute walk distance in CONFIRM-HF and FAIR-HF (57, 59). Most recently, AFFIRM-AHF demonstrated that when used in patients hospitalized for acute HF, IV FCM was associated with a decrease in HF hospitalizations, but did not reduce CV death; and HEART-FID noted no significant difference in their primary outcome- a composite of death, hospitalizations for HF, or change in 6-minute walk distance- with the use of IV FCM in ambulatory patients with HFrEF (58, 65). Both studies note the challenge of interpreting results given the inherent heterogeneity of HF patient populations included, and the need to clarify criteria for iron-deficiency to identify which patients may benefit most (58, 65). Nevertheless, based on the demonstrated safety and potential to improve functional status and quality of life, IV iron supplementation is a Class IIa recommendation for use in patients with HF with iron deficiency (17, 66).

Another important intervention shown to improve quantity and QOL in patients with HF is exercise training, which carries a Class Ia level of recommendation for management of patients with Stage C HF regardless of EF (17). Led by the HF-ACTION trial, which demonstrated that exercise training was safe and carried modest but significant reductions in all-cause mortality or hospitalization and cardiovascular mortality or HF hospitalization, there are numerous additional cardiac rehabilitation studies demonstrating improvement in functional capacity and QOL in the HF population (60–62). Most relevant studies focused on the HFrEF population, but available data suggest a benefit in the HFpEF subset as well, and exercise training should be prescribed for all HF patients who lack a contraindication (60–62, 67).

In addition to these HF-focused interventions, it is important to take a broader and holistic approach to optimization of the care of patients with HF, including consideration of socioeconomic factors and comorbidities that may contribute to HF exacerbations and hospitalizations (17, 68, 69). Consideration of access to food that is low in sodium, transportation to visits, access to medications that are part of GDMT for HF, and evaluating and supporting health literacy are all essential components of reducing disparities in HF care and outcomes that are based in social determinants of health (70). Counseling patients on smoking and alcohol cessation, screening for and treating sleep disorders including obstructive sleep apnea, achieving optimal glycemic control, and enhancing BP management are all important components to prevent recurrence of decompensation in patients with HF (17). Lastly, depression is a comorbid condition commonly plaguing patients with HF and increasing the risk for rehospitalization and all-cause mortality (71). The prevalence of depression in patients with HF is higher than in the general population and limits the ability to perform needed self-care activities (71). Trials have produced little evidence of benefits of psychoactive medications on HF outcome (71). However, in contrast, a meta-analysis of the use of exercise training in HF showed it to be consistently associated with improvement in symptoms of depression, thus providing another reason to encourage increased activity for patients with HF (72).

## Future directions

Despite numerous advancements in treatment strategies and therapies, episodes of decompensation remain a detriment to patients with HF and a significant healthcare burden. The goals of outpatient treatment of decompensated HF are to prevent hospitalization and return patients to previous or better clinical status (38). Systems of care that successfully manage decompensated HF in the ambulatory setting include interventions that promote early identification of an episode of decompensation, facilitate timely and appropriate intervention, provide intensification of HF care until resolution of the episode, and then focus on optimization of patients with HF to reduce the risk of future decompensation.

While there have been transformational trials yielding dramatically improved prognosis associated with the use of GDMT and other areas of HF care, significant work is needed to improve access to these therapies and reduce disparities in HF care. Cost, insurance approval, disparities in accessing care related to other social determinants of health, as well as logistics of navigating the healthcare system present barriers to utilization of GDMT, IV FCM, outpatient IV diuretics, and exercise training for patients with HFpEF, despite their potential to improve care and reduce the burden of disease (9, 38, 53, 56, 69, 70, 73). Addressing these factors is of utmost importance given the growing body of evidence demonstrating the impact of socioeconomic status and social determinants of health on cardiovascular outcomes in general and HF outcomes specifically (68–70).

Additionally, sex and race-based disparities with underrepresentation in trials for GDMT and RPM has led to gaps in understanding of potential unique risks and benefits and there has also been under-referral for RPM noted in these populations as well, despite the benefit noted specifically for these populations (24, 74).

Some of the potential interventions aimed at reducing disparities and increasing utilization of therapies for HF include utilizing integrated electronic medical record alerts, multidisciplinary teams with workforce and leadership diversity and clinician education and feedback, which have shown promise in increasing utilization of best practices in HF care and warrant ongoing investigation (26, 63, 64, 75, 76). Ensuring policies are in place that encourage enrollment of women and historically underrepresented populations is critical to improve understanding of the unique benefit of medications, invasive and noninvasive remote monitoring and other interventions aimed at improving outcomes for patients with HF (70, 74).

There is an ongoing opportunity to harvest the benefits of novel technology to improve HF care. The area of artificial intelligence holds great promise with the ability to create multiparameter models with input from invasive and noninvasive sources, that will allow HF care to evolve from identifying decompensation when it occurs to being able to predict and intervene earlier and with greater success (20, 26, 77, 78). Improved understanding of a patient's HF risk profile would allow low risk patients to be successfully managed in the ambulatory setting and would facilitate appropriate allocation of resources to those at highest risk. Related to this is the opportunity to optimize integration of relevant data from RM devices, wearable devices, and testing into the EMR, to allow efficient, standardized and appropriate care for patients with HF, particularly those in evolving decompensation (20, 79, 80).

Lastly, there is continued focus on providing acute HF care outside the hospital environment. While some aspects of this are increasingly available, including outpatient IV and SQ diuretics, and point of care tools like ultrasound and laboratory tests to augment diagnosis and decision making, continued work is needed to determine optimal timing and patient selection and also ensure these important events are recognized with the same prognostic weight as HF hospitalizations (10, 35, 38, 81).
